# Effects of antioxidants on diabetic kidney diseases: mechanistic interpretations and clinical assessment

**DOI:** 10.1186/s13020-022-00700-w

**Published:** 2023-01-09

**Authors:** Yuting Sun, De Jin, Ziwei Zhang, Yuehong Zhang, Yuqing Zhang, Xiaomin Kang, Linlin Jiang, Xiaolin Tong, Fengmei Lian

**Affiliations:** 1 Guang’anmen Hospital, China Academy of Chinese Medical Sciences, Beixiange 5, Xicheng District, Beijing, 100053 China; 2 Hangzhou Hospital of Traditional Chinese Medicine, Hangzhou, China; 3 College of Chinese Medicine, Changchun University of Chinese Medicine, ChangchunJilin, 130117 China; 4 Institute of Metabolic Diseases, Guang’anmen Hospital, China Academy of Chinese Medical Sciences, Beijing, China

**Keywords:** Diabetic kidney disease, Antioxidants, Meta-analysis, Systematic review, Randomized controlled trials, Mechanistic interpretations, Clinical assessment

## Abstract

Diabetic kidney disease (DKD) is more prevalent with an increase in diabetes mellitus. Oxidative stress is a major factor in the occurrence and progression of DKD. Defending against oxidative stress and restoring antioxidant defense might be key to preventing and treating DKD. The purpose of this article is to provide an explanation of how oxidative stress affects DKD, conduct a systematic review and meta-analysis on DKD, and examine the effect of antioxidants on the disease. An analysis of 19 randomized controlled trials showed that the use of antioxidants could reduce UAE (albumin excretion rate) in patients with DKD (SMD: − 0.31; 95% CI [− 0.47, − 0.14], I^2^ = 0%), UACR (urine albumin/creatinine ratio) (SMD: − 0.60; 95% CI [− 1.15, − 0.06], I^2^ = 89%), glycosylated hemoglobin (hbA1c) (MD: − 0.61; 95% CI [− 1.00, − 0.21], I^2^ = 93%) and MDA (malonaldehyde) (SMD:-1.05; 95% CI [− 1.87, − 0.23], I^2^ = 94%), suggesting that antioxidants seemed to have therapeutic effects in patients with DKD, especially in reducing proteinuria and hbA1c. The purpose of this study is to provide new targets and ideas for drug research and clinical treatment of DKD.

**Supplementary Information:**

The online version contains supplementary material available at 10.1186/s13020-022-00700-w.

## Introduction

There is a direct correlation between diabetes and diabetic kidney disease (DKD) prevalence worldwide [1]. Diabetes is estimated to affect 642 million people worldwide by the year 2040. The main cause of chronic kidney disease (CKD) and end-stage renal disease (ESRD) is DKD, which is progressive and irreversible renal damage [2]. Microalbuminuria and tubulointerstitial fibrosis are two of the common microvascular complications of diabetes [3, 4]. Type 2 diabetes mellitus accounts for 5–40% of cases of DKD, which has been linked to several structural alterations in the kidneys [5]. Glomerular basement membrane thickening (formed by the parallel connection of capillary and tubular capillaries, basement membrane thickening) occurred successively [6–8]. Changes in glomerulus mainly included the loss of endothelial window, expansion of mesangial matrix, podocyte deletion, and podocyte disappearance [9], and continued to develop segmental mesangial dilatation [10, 11]. With persistent proteinuria associated with hypertension, glomerular filtration rate (GFR) declines due to glomerular hyperfiltration [12]. As GFR declines in DKD, both renal and non-renal complications occur, and anemia appears earlier than in other types of CKD [13].

## Oxidative stress in diabetic kidney disease

### Mechanisms by which oxidative stress is involved in DKD

In diabetic vascular complications, oxidation plays an important role [13–16]. Hyperglycemia, oxidative stress, and diabetic complications are closely related, according to numerous studies [17, 18]. The occurrence and development of DKD involve a variety of pathways and mediators, which is a disease with complex pathogenesis. Abnormal homeostasis, including metabolic disorders, hormone synthesis, and hemodynamic abnormalities, contribute to the pathogenesis of DKD [19]. A large amount of oxidative stress occurs in patients with type 2 diabetes, resulting in complications, and the increase in oxidants comes from mitochondria that are not functioning and NOX1 (NADPH oxidase 1) in the liver [20, 21]. Hyperglycemia leads to the activation of the pathways and the production of reactive oxygen species, and the increase of cytokines and chemokines such as IL-6 (interleukin 6), MCP-1 (monocyte chemoattractant protein-1), TGF-β (transforming growth factor-β), and VEGF (vascular endothelial growth factor) leads to inflammation, fibrosis, and increased vascular permeability [22]. Pathogenesis of DKD is influenced by a number of factors in series. Nicotinamide adenine dinucleotide phosphate (NADPH) oxidase increases NADPH oxidase, and NAPDH oxidase increases ROS (reactive oxygen species). Increased ROS production leads to a continuous increase in TGF-β, which promotes the process of renal fibrosis in the renal tubulointerstitial [23]. Damage from Ang-II is caused by oxidative stress, which is caused by the RAAS (Renin–angiotensin–aldosterone system).

Inflammatory response plays an important role in the pathogenesis of DKD [19]. Hyperglycemia leads to the expression of inflammatory mediators, which in turn promotes mesangial proliferation, podocyte injury, tubular injury, and leukocyte infiltration, resulting in varying degrees of renal injury [24, 25]. Inflammatory factors induce vascular remodeling, endothelial cell dysfunction, extracellular matrix deposition, mesangial proliferation, podocyte, and tubular death, and in addition, glomerular basement membrane (GBM) thickening, and glomerulosclerosis are hallmarks of DKD [9, 26]. The role of many inflammatory factors in the pathogenesis of DKD has been confirmed, NF-κB (nuclear factor κB) regulates inflammatory cytokines, chemokines, and cell adhesion proteins to damage the renal function of DKD [27]. Different polymorphisms of IL-6 play an important role in DKD patients [28], and urinary IL-6 levels are often increased in DKD patients with poor prognosis of renal function [29]. TNF-α (tumor necrosis factor-alpha) aggravates DKD-related inflammatory responses by affecting the recruitment and activation of leukocytes [24, 30]. TNF-α may damage a variety of cells such as renal epithelial cells, endothelial cells, mesangial cells, and podocytes [29–31]. Chemokines are also released as a result of inflammation-induced cytokine stimulation. Patients with DKD have elevated levels of chemokines and chemokine receptors in their kidneys [32, 33], and their cognate receptors are expressed by podocytes and tubular cells [34]. In DKD, cell adhesion molecules (CAMs) play an important role in the interaction between leukocytes and endothelial cells [35]. During DKD pathogenesis, inflammation and oxidative stress are closely linked, and these two factors are dependent on one another [3, 35–38]. In mesangial cells, increased TNF-α promotes oxidative stress through NADPH activation [27]. The direct cascade between inflammation and oxidative stress involves p38 MAPK (mitogen-activated protein kinase) and transcription factor activator protein 1 (AP-1) and c-Jun N-terminal kinase (JNK) [38]. Oxidative stress, inflammation, and DKD progression are also primarily caused by intracellular and extracellular oxygen-derived free radicals and inflammation [39]. Increased ROS production after NF-κB activation contributes to inflammation in DKD. The excessive production of ROS plays an important role in the pathogenesis of DKD by activating NF-κB and inflammatory cytokines [39, 40].

**Fig. 1:**
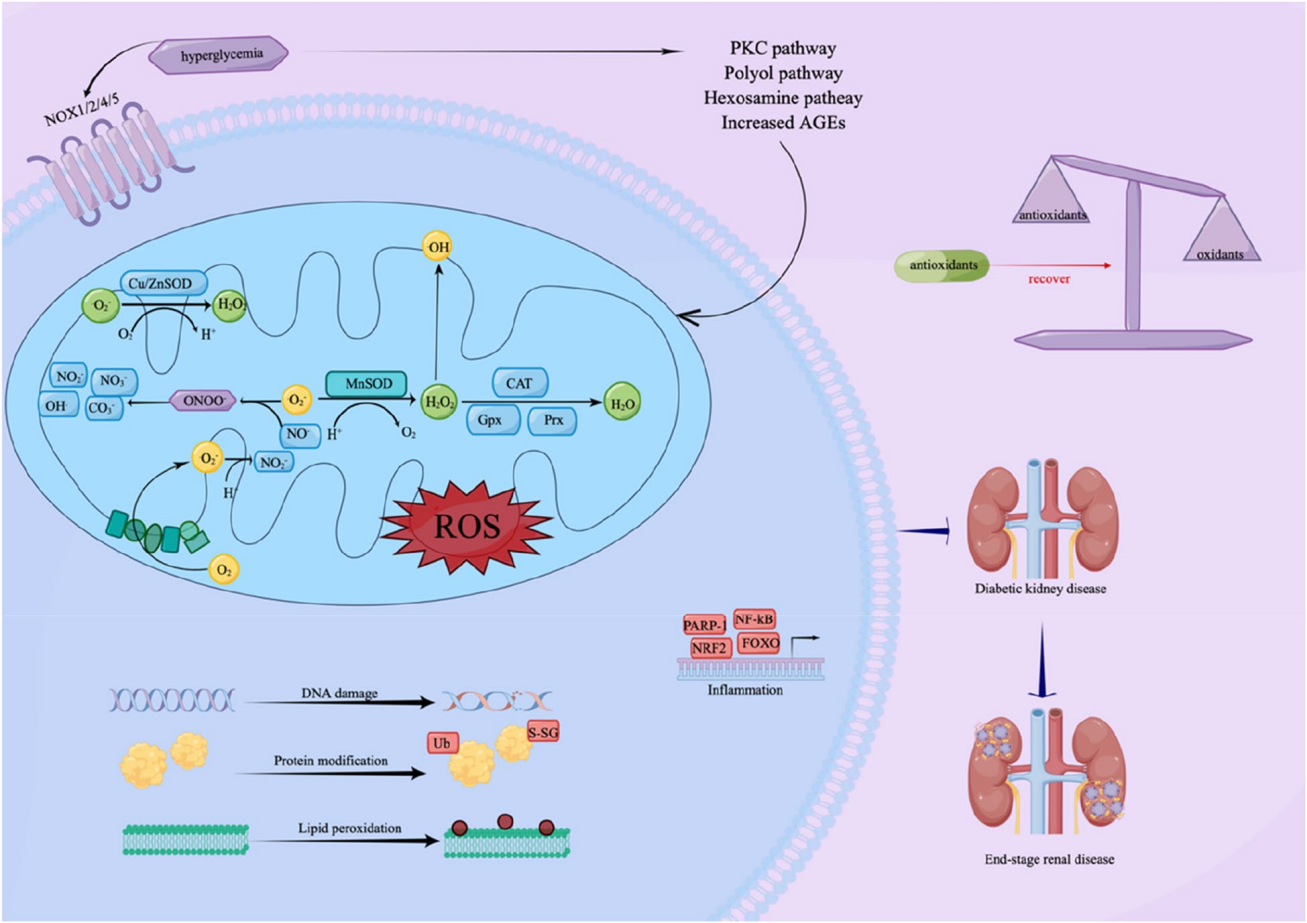
Oxidative stress is involved in the pathogenesis of DKD. Copper/zinc superoxide dismutase (Cu/ZnSOD) and manganese superoxide dismutase (MnSOD) catalyze the mutation (or distribution) of superoxide (O_2_^• −^) radicals to hydrogen peroxide (H_2_0_2_) in the mitochondrial membrane space (IMS) and matrix, respectively. Hydrogen peroxide (H_2_O_2_) is converted to water by catalase (CAT) and a group of glutathione peroxidases (gpx) and peroxide reductases (Prxs). H_2_O_2_ spreads easily to other parts of the mitochondria or cytoplasm. O_2_—reacts with nitric oxide (NO•) to produce peroxynitrite (ONOO-), ONOO- decomposes into highly oxidized intermediates such as NO_2_ ^−^ , OH•, CO_3_ ^−^ , etc., and finally forms stable NO_3_ ^−^ . NF-κB, nuclear factor κB; Nrf2, nuclear factor (erythroid-derived 2)-like 2; PARP-1, poly (ADP-ribose) polymerases; FOXO, forkhead box protein O. ROS, reactive oxygen species

The imbalance between oxidative and antioxidative systems can cause oxidative stress in several pathological states, including diabetes-induced cell damage [41, 42]. It is thought that hyperglycemia promotes oxidative stress both enzymatically and nonenzymatically [43, 44]. ROS, which is hazardous to cells, especially the cell membrane, is produced because of oxidative stress [43]. In hyperglycemia, aldose reductase inhibits the expression of antioxidant enzymes, including superoxide dismutase and glutathione peroxidase [45]. Diabetic long-term complications are associated with hyperglycemia caused by increased production of reactive oxygen species and attenuated scavenging enzymes [44, 45]. Multiple mitochondria in the kidney make it more susceptible to damage caused by OS (overall survival) [46]. Hyperglycemia can lead to the massive production of ROS [47, 48], when excessive ROS is produced, the antioxidant enzyme system reaches saturation, and excess ROS interacts with membranes, lipids, proteins, enzymes and DNA, resulting in cell damage and dysfunction [49], especially vascular and endothelial function [50–52], free radicals cause oxidative damage to the kidney, and enhance fibrosis, cell proliferation, and matrix accumulation [51, 53], ROS reduces the bioavailability of nitric oxide (NO) and affects the medium filtration of glomeruli [51]. Meanwhile, hyperglycemia produces AGEs (advanced glycation end products), PKC (protein kinase C), free fatty acids, and cytokines, thereby activating the NADPH oxidation system in renal cells [54, 55]. DKD is therefore characterized by excessive production of ROS and destruction of antioxidant defense mechanisms [54]. ROS plays a role in kidney inflammation and renal fibrosis that contribute to the progression of DKD. The production of ROS induced by hyperglycemia stimulates the recruitment of numerous inflammatory cells and the production of inflammatory cytokines, growth factors, and transcription factors related to the pathological process of DKD [55]. Increased ROS production leads to the recruitment of ECM- (extracellular matrix-) producing cells along with the activation of fibrogenic factors such as TGF-β and connective tissue growth factor (CTGF), thereby promoting the progression of renal fibrosis and sclerosis [56]. Sources of ROS production such as nitric oxide synthase uncoupling, glycolysis, xanthine oxidase, reduced NADPH oxidases, and advanced glycation end products are considered potential pathogenesis of DKD [57]. Meanwhile, the progression of DKD to ESRD may be influenced by oxidative stress [56, 57]. Antioxidants inhibit extracellular matrix (ECM) synthesis of mesangial cell proteins induced by high glucose, prevent glomerular hypertrophy, reduce proteinuria, and reduce the expression of transforming growth factor-β1 (TGF-β1) and ECM in glomeruli of DKD animals, which is a function of oxidative stress in DKD [58–60]. It has been shown that ROS may play an important role in DKD as a signaling molecule [61, 62] (Fig. 1).

### Antioxidants in diabetic kidney diseases

Antioxidants in the clinic have anti-aging, anti-cancer, anti-cataract, antidiabetics, anti-inflammatory, and antibacterial effects, play a critical role in the treatment of cardiovascular disease, and have the function of hepatoprotective, nephroprotective, and neuroprotective. A growing number of studies from animal models and human DKD patients have shown the positive effects of antioxidants on DKD through different molecular mechanisms. As a key regulator protector of antioxidants and cells, Nrf2 (nuclear factor (erythroid-derived 2)-like 2) is mainly activated in response to oxidative stress [63, 64]. Fufang-Zhenzhu-Tiaozhi (FTZ), with oxidative stress effect, experiments showed that the protein expression of oxidative stress factors HO-1 (hemoglobin oxygenase-1 (hypocretin-1)), NQO1 (Quinone oxidoreductase (NAD(P)H Quinone Dehydrogenase 1)) and Nrf2 was downregulated in the DKD model, and the protein expression of HO-1, NQO1, and Nrf2 in kidney tissues of the FTZ group was upregulated [65]. In STZ-induced rat DKD models, serum MDA levels were significantly increased, CAT (catalase), SOD (superoxide dismutase), and GPx (glutathione peroxidase) activities were significantly reduced, and Notoginsenoside R1 (NR1) could upregulate α3β1 integrin, reduce serum MDA levels, and increase CAT, SOD and GPx activities [66]. In HFD (high-fat diet) and STZ- (streptozotocin-) induced DKD rat models, hydroxyl safflower yellow A (HSYA) increased SOD and GSH-Px (glutathione) levels, reduced MDA in serum and renal tissue and protected renal function [67]. Hyperglycemia-mediated PKC-β overexpression leads to NADPH oxidase activation and ROS production. PKC-β inhibitor Ruboxistaurin reduces proteinuria in animal and human models [50, 68]. The interaction of AGE and RAGE (receptor for advanced glycosylation end products) in DKD activates the expression of nuclear factor-k, which can stimulate ROS production. In DKD models, Pyridoxamine was shown to inhibit the Maillard response, blocking protein glycosylation and AGE product deposition [69]. Nuclear factor erythroid 2-related factor 2 (NFE2L2, Other aliases include Nrf2 and HEBP1) is a transcription factor that prevents oxidative stress and injury [70–73]. Heme oxygenase-1 (HMOX1), as a target gene of NFE2L2, plays an important role in antioxidant resistance [74, 75]. The 7th member of SLC30 family (SLC30A7) exerts antioxidant effects in high-glucose-induced cells through the NFE2L2/HMOX1 signaling pathway [76]. High levels of glucose and increased ROS production over-activate sodium/glucose co-transporter type 2 (SGLT2) transporters in tubular cells, which in turn can exacerbate oxidative stress. SGLT2 inhibitors show a positive significance for DKD through a beneficial balance between oxidative and antioxidant mechanisms [77]. Studies have shown that oxidative stress and nuclear transcription factor specificity protein 1 (Sp1) are closely related to the pathogenesis of DKD [78, 79]. The TLR4 (toll-like receptor 4) /NF-κB signaling pathway may be an upstream pathway for PGC-1α (proliferator-activated receptor γ coactivator-1α) by regulating mitochondrial-associated oxidative damage and promoting DKD tubular damage [80]. GCN5L1- (general control of amino acid synthesis 5-like 1-) mediated MnSOD (manganese superoxide dismutase) acetylation exacerbates renal damage from oxidative stress [81]. MaR1 can mitigate DKD through the LGR6—(leucine-rich repeat domain-containing G protein-coupled receptor 6 -) mediated cAMP—(cyclic adenosine 3’,5’—monophosphate-) SOD2 (Mn-SOD) antioxidant pathway [82].

Oxidation-antioxidant system imbalance can lead to tissue damage [83]. Studies have shown that restoring the balance between oxidative stress and antioxidant defenses may be a potential drug target for DKD prevention and treatment [84]. CuNPs (hydrogen sulfide) have antioxidant properties and are beneficial for diabetes [85]. Acridine and phenan derivatives were found to scavenge free radicals and to have anti-diabetic properties. An antidiabetic activity has been observed in Kuning ethyl acetate extract by scavenging the DPPH free radical and superoxide anion [86]. Proteinuria can be corrected by propyl gallate by reducing endothelial cell proliferation, the pathological changes to the glomeruli, and improving endothelial cell proliferation [87]. The extracts of Diospyros lotus seeds have anti-lipid peroxidation and hydrogen peroxide free radical scavenging effects and are protective against renal injury [88]. By reducing ROS and oxidative damage to the kidneys, vitamin C maintains kidney function [86]. For the clinical application of antioxidants in the treatment of DKD, drugs that remove O2• − and H2O2 from the intracellular space and mitochondrial matrix may have positive implications. SOD, SOD-Catalase, and GPX mimic may be effective for DKD [84]. There is evidence that NaHS can reverse biochemical, apoptosis, oxidative stress, and pathological parameters in DKD mice [62]. Continuous selenium therapy for 12 weeks has reportedly been shown to drastically lower insulin levels in DKD patients [89]. Resveratrol is a natural antioxidant. Experimental studies have shown that oral resveratrol can improve the level of creatinine clearance and inflammatory markers, and significantly increase SOD, CAT, GSH-Px, and glutathione S transferase (GST) in diabetic patients and diabetic mouse models [90, 91]. Studies have shown that diabetic rats can reduce proteinuria after curcumin treatment [92]. Through Nrf2 and Adenosine-activated protein kinase, curcumin reduces the pathophysiological changes of DKD and the OS of glomeruli. Antioxidants have also been shown to benefit DKD patients in many clinical studies. In 18 patients with DKD, antioxidant enzyme activity was changed, and redox status was affected [93, 94]. In addition to participating in oxidative defense and immune functions, selenium is an essential trace element. According to the study, fat-soluble vitamins improve renal injury, inflammation, and OS in patients with DKD. Antioxidants have been found to have positive effects on DKD in many studies, but their effectiveness needs to be systematically examined because of limitations like small sample sizes.

## Clinical assessment

Systematic review and Meta-analysis were used to analyze and evaluate the treatment of DKD with antioxidants.

## Methods

### Search strategy and study selection

CNKI (China national knowledge infrastructure), Wanfang Database, PubMed, and Cochrane Library databases were searched from the establishment of the database to October 22, 2022. Languages are limited to Chinese and English. We also searched online clinical trial registries such as ClinicalTrials.gov (Clinical trials.gov/) and the World Health Organization's International Clinical Trials Registry Platform (www.who.int/ictrp). The specific search strategy depends on the specific database. The authors list Pubmed search strategies. The program is registered with PROSPERO as CRD42021297266.

The full electronic search strategy for PubMed was provided in File 1 according to the search history (Additional file 1: File S1).

1. Types of studies: only RCTs (randomized controlled trials) were eligible for this review. Consider only double-blind, placebo-controlled trials.
2. Types of participants: Patients with type 1 and type 2 diabetes with DKD (with albuminuria).
3. Types of interventions: Any antioxidants supplement (including but not limited to vitamin C, vitamin E, Se, Zinc, green tea, resveratrol, melatonin, coenzyme Q10, and crocin) should be used alone or in combination.
4. Types of outcomes: Primary outcome is the UAE. Secondary outcomes included UACR, Serum creatinine (SCr), hbA1c, and MDA.
5. Safety outcomes included adverse events.

### Data collection and analysis

Collected documents are processed using document management software as references. Two independent auditors (SYT and JD) check the results against the inspection criteria, and then the auditors check each other. Any disagreements are discussed with the third reviewer (ZZW). Data extraction Table 1 are used to extract data and have the following requirements. (1) The basic characteristics of the included literature, literature name, publication year, literature source, author, and so on. (2) Research methods, study design, random method, allocation hiding method, blind method, duration, etc. (3) Basic characteristics of participants included in the study, including the number of participants, age, gender, etc. (4) Intervention and control methods during clinical trials, including intervention methods, number of patients, administration route, dose, time, course of treatment, follow-up, etc. (5) The outcome index of the study, the index measurement method, and the data statistical analysis method used in the experiment. (6) The results.

**Table 1:** Summarize the main features and findings of the included studies

Study	Study population	Participants (Male/Female)	Age	Intervention Antioxidants	Antioxidants dose	Control	Treatment duration	Outcomes	Adverse Events	Notes
Gaede et al. (2001) [95]	T2DM who have stable HbA1c control (no more than 10% change over the previous 2 months)	N:29	58.7 ± 7.3	Vitamin C + Vitamin E	Vitamin C (1250 mg/d) + Vitamin E (680 IU/d)	Placebo	4 weeks	UAE SCr		Double-blind
Farvid et al. (2005) [96]	Diabetes for at least 1 year, with a bias toward those who were not macroalbuminuric and hypertensive	N:76 P:M(9) F(10) M:M(8) F(10) V:M(9) F(11) MV:M(9) F(10)	P:50 ± 9 M:52 ± 8 V:50 ± 9 MV:50 ± 9	M (Zinc sulphate + magnesium oxide) V (Vitamin C + Vitamin E) MV (Zinc sulphate + magnesium oxide + Vitamin C + Vitamin E)	M (Zinc sulphate 15 mg + magnesium oxide 100 mg) V (Vitamin C 100 mg + Vitamin E 50 IU); MV (Zinc sulphate 15 mg + magnesium oxide 100 mg + Vitamin C 100 mg + Vitamin E 50 IU)	Placebo	3 months	UAE		Double-blind
Giannini et al. (2007) [89]	IDDM patients with microalbuminuria	N = 20	18.87 ± 2.91	Vitamin E	1200 mg/day	Placebo	6 months	UAE		Double-blind
Parham et al. (2008) [92]	NIDDM patients with microalbuminuria	N:42 I:M(62%) F(38%) C:M(52%) F(48%)	I:52.0 ± 9.3 C:54.5 ± 9.2	Zinc	30 mg/day	Placebo	3 months	UAE HbA1c		Double-blind
Khajehdehi, et al. (2011) [97]	Type 2 diabetic nephropathy (proteinuria ≥ 500 mg/day)	N:40 I:M(9) F(11) C:M(13) F(7)	I:52.9 ± 9.2 C:52.6 ± 9.7	Turmeric	Each meal containing 500 mg	Placebo	2 months	SCr UAE UACR		Double-blind
Fallahzadeh, et al. (2012) [98]	Patients with type 2 diabetes with macroalbuminuria (urinary albumin excretion 300 mg/24 h)	N:60 I:M(15) F(15) C:M(13) F(17)	I:55.9 ± 8.3 C:57.6 ± 7.5	Silymarin	420 mg/d	Placebo	3 months	SCr HbA1c MDA UACR	I:7 C:2	Double-blind
Noori et al. (2013) [99]	Patients with type II diabetes	N:34 I:M(7) F(10) C:M(6) F(11)	I:60.0 ± 2.0 C:61.0 ± 3.0	Lipoic acid and pyridoxine	lipoic acid 800 m/d and pyridoxine 80 mg/d	Placebo	12 weeks	MDA UAE		Double-blind
Haghighat et al. (2014) [90]	Patients with T2DM and FBS > 126 mg/dl	N:45 I:M(5) F(18) C:M(7) F(15)	I:55.9 ± 5.9 C:55.2 ± 5.6	Tocotrienol -enriched canola oil	200 mg/day	pure canola oil	8 weeks	UAE		Double-blind
Zhu et al. (2016) [93]	Patients with T2D	N:160	I:56.5 ± 9.8 C:57.3 ± 10.3	Telmisartan + probucol	500 mg/dose	Telmisartan	24 weeks	SCr HbA1c UAE	I:5 C:5	Double-blind
Bahmani et al. (2016) [100]	Diabetic renal disease with proteinuria level of > 0·3 g/24 h	N:60 I:30 C:30	40–85	Se supplements	200 µg/d	Placebo	12 weeks	MDA HbA1c		Double-blind
Borges et al. (2016) [101]	DM type 1 or 2, and had persistent micro- or macroalbuminuria	N:47 I:M(11)F(12) C:M(16) F(8)	I:63(60–65) C:59(49–63)	ACE inhibitors and/or ARBs plus GTP	maximum dose (corresponding to 800 mg of EGCG)	ACE inhibitors and/or ARBs plus placebo	12 weeks	UACR UAE HbA1c	I:2 C:1	Double-blind
Elbarbary et al. (2018) [102]	Diabetic renal disease with a proteinuria level > 0.3 g/24 h	N:50 I:M(8) F(17) C:M(8) F(17)	I:61.1 ± 11.3 C:61.6 ± 10.1	Coenzyme Q10	100 mg/day	placebo	12 weeks	UAE HbA1c UACR		Double-blind
Aghadavod et al. (2018) [103]	Patients with diabetic nephropathy, despite oral angiotensin-converting enzyme inhibitors	N:90 I:M(20) F(25) C:M(23) F(22)	I:12.4 ± 3.4 C:13.3 ± 2.8	Carnosine	500 mg	Placebo	3 months	HbA1c UACR SCr MDA		Double-blind
Gholnari et al. (2018) [104]	T2DM and referred to a diabetes clinic with newly diagnosed confirmed albuminuria were evaluated or inclusion in the study	N:60 I:M(14) F(16) C:M(13) F(17)	I:56.8 ± 9.7 C:55.7 ± 10.8	Resveratrol	500 mg/day	Placebo	90 days	UACR HbA1c SCr MDA		Double-blind
Tan et al. (2019) [105]	Patients with DN	N:54 I:M(8) F(19) C:M(9) F(19)	I:62.2 ± 9.8 C:64.5 ± 9.2	Vitamin E	800 mg/d	Placebo	12 weeks	HbA1c		Double-blind
Sattarinezhad et al. (2019) [106]	T2DM who have stable HbA1c control (no more than 10% change over the previous 2 months)	N:54 I:M(18) F(9) C:M(17) F(10)	I:59 ± 10 C:62.8 ± 11.6	Tocotrienol-rich vitamin E	Tocovid 200 mg	Placebo	12 weeks	SCr HbA1c MDA	I:13 C:15	Double-blind
Satari, et al. (2021) [107]	Diabetic patients who are reviewed on a regular basis	N:59 I:M(20) F(11) C:M(18) F(10)	I:66(13) C:70(13)	Tocotrienol-rich vitamin E	200 mg/twice daily	Placebo	12 months	UACR HbA1c	I:3	Double-blind
Koay et al. (2021) [94]	DN, glomerular filtration rate 15 to 89 mL/minute/1.73m2, moderate blood pressure	N:46 I:M(13) F(9) C:M(14) F(10)	I:66.9 ± 6.9 C:64.3 ± 7.7	Melatonin	10 mg/d	Placebo	12 weeks	MDA		Double-blind
Jaafarinia et al. (2022) [108]	Patients aged ≥ 18 years with T2DM	N:40 I:M(12) F(9) C:M(11) F(8)	I:63.86 ± 10.62 C:62.68 ± 9.84	crocin	15 mg	Placebo	90 days	HbA1c UACR UAE SCr	I:1 C:1	Triple-blind

*I* intervention groups, *C* control groups, *UAE* Albumin excretion rate, *UACR* Urine albumin/creatinine ratio, *SCr* Serum creatinine, *hbA1c* glycated hemoglobin glycosylated hemoglobin, *MDA* Malonaldehyde

The statistical analysis of this paper is carried out by two reviewers independently using Cochrane collaborative review management software (RevMan5.4). Publication bias was funnel plot test. Publication bias is measured only when a subgroup contains ten or more studies.

### Assessment of risk of bias

The risk assessment is conducted according to Cochrane renal group, and the selection bias is evaluated by random sequence generation and promotion, the performance bias is evaluated by blinding of investigators and participants, the detection bias is evaluated by blinding of outcome assessors, the attrition bias is evaluated by incomplete outcome data, the reporting bias is evaluated by selective reporting and possibly other sources of bias.

### Measures of treatment effect

Mean difference (MD) or standard mean difference (SMD) were used to evaluate the effect of the intervention on continuous variables. Random effects models were used to summarize the data. The classification results were expressed as a 95% CI.

### Assessment of heterogeneity

Heterogeneity was evaluated by the Chi^2^ test of N-1 degree of freedom, and the difference was statistically significant when the alpha value was 0.05, Cochrane-I^2^. I^2^ value of 25% represents low-level heterogeneity, I^2^ value of 50% represents medium heterogeneity, and I^2^ value of 75% represents high-level heterogeneity. Results with high heterogeneity were subgroup analyzed to explore the source of heterogeneity.

## Results

During this period, a total of 4191 related kinds of literature were searched, excluding duplicate publications, reviews, systematic reviews, meta-analyses, and non-RCTs, leaving 554 RCTs. Due to incomplete data in some pieces of literature, non-double-blind, and placebo-controlled trials, 19 studies [89, 90, 92–108] finally met the inclusion criteria. The filtering process is shown in Table 2.

**Table 2:**
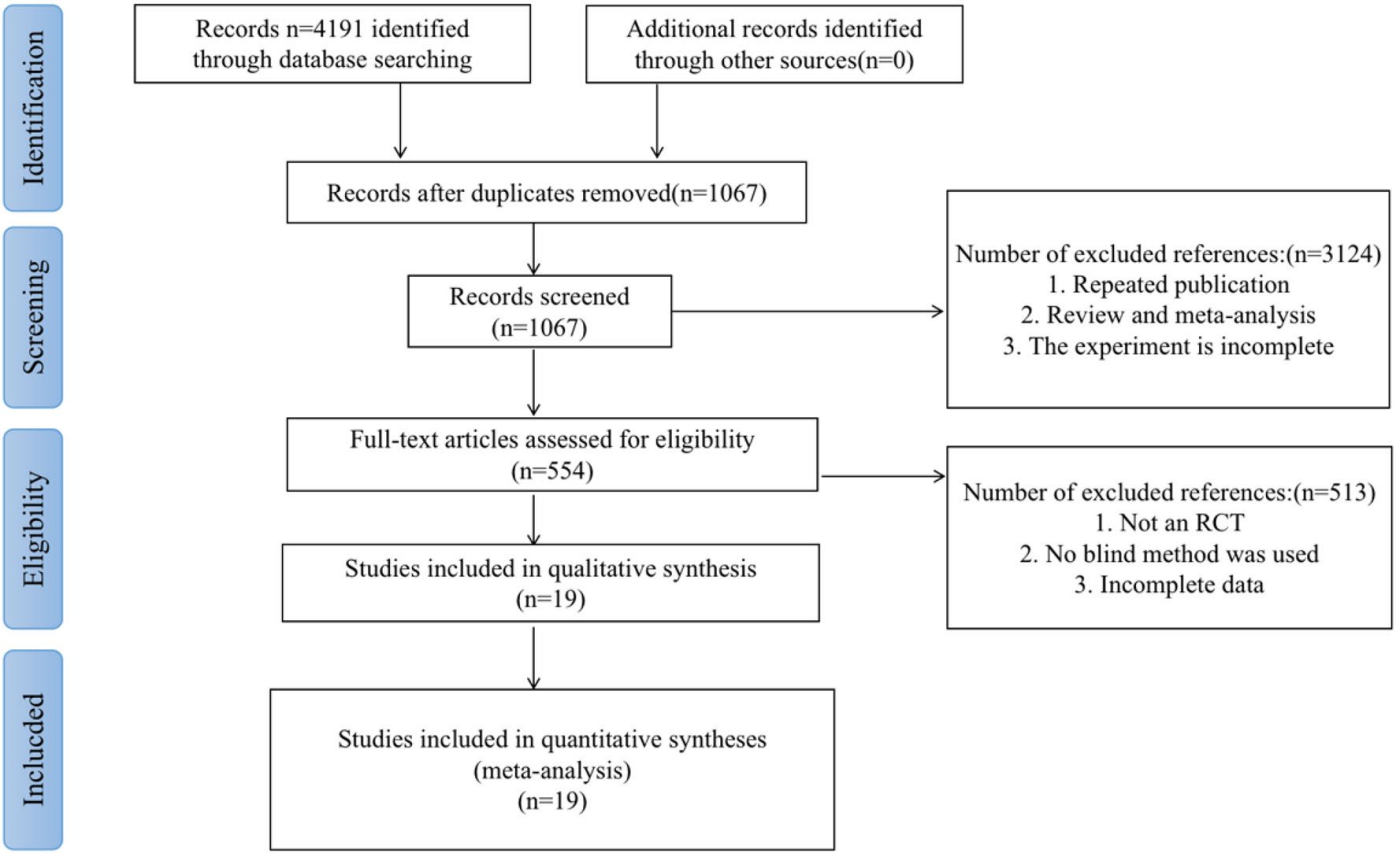
The filtering process

### Primary outcomes

**Fig. 2:**
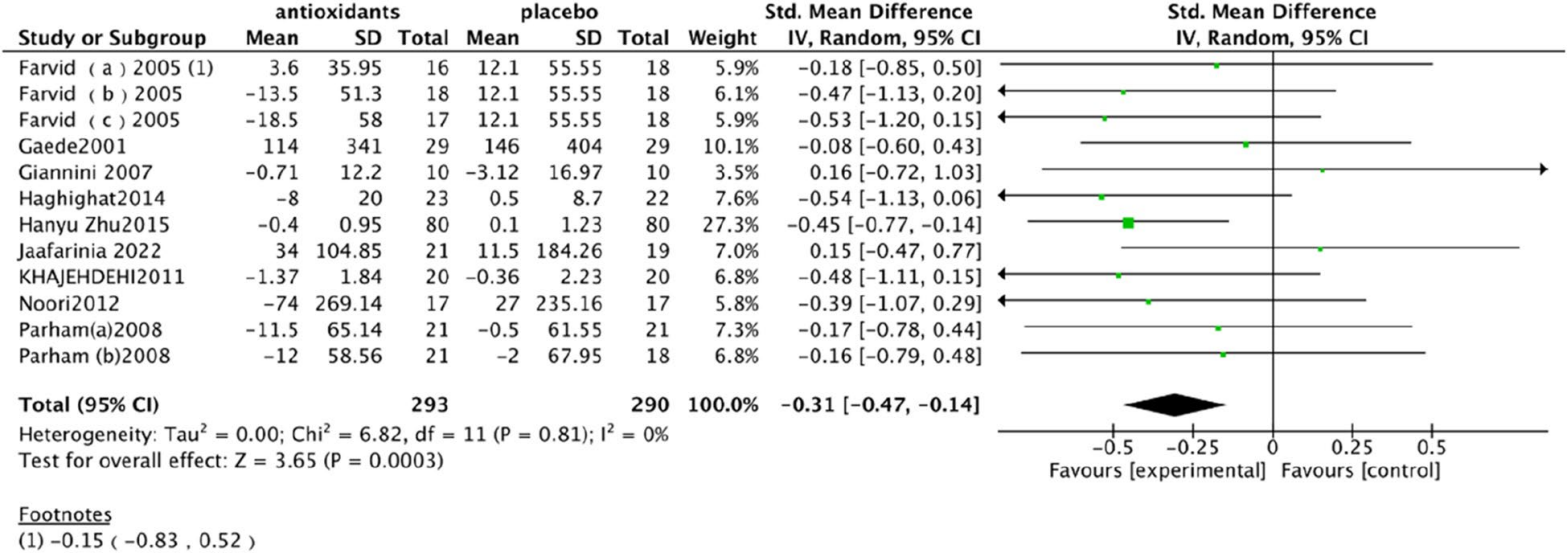
Effect of antioxidants vs. control on albumin excretion rate (UAE)

Albumin excretion rate (UAE). In a pooled analysis of 9 studies [89, 90, 92, 93, 95–97, 99, 108], the use of antioxidants was associated with a significant reduction in UAE levels compared with placebo (SMD: − 0.31; 95% CI [− 0.47, − 0.14], Fig. 2). The heterogeneity of this analysis was low (Chi^2^ = 6.82, df = 11 (P = 0.81); I^2^ = 0%). The Test for overall effect: Z: 3.65 (P = 0.0003).

**Fig. 3:**
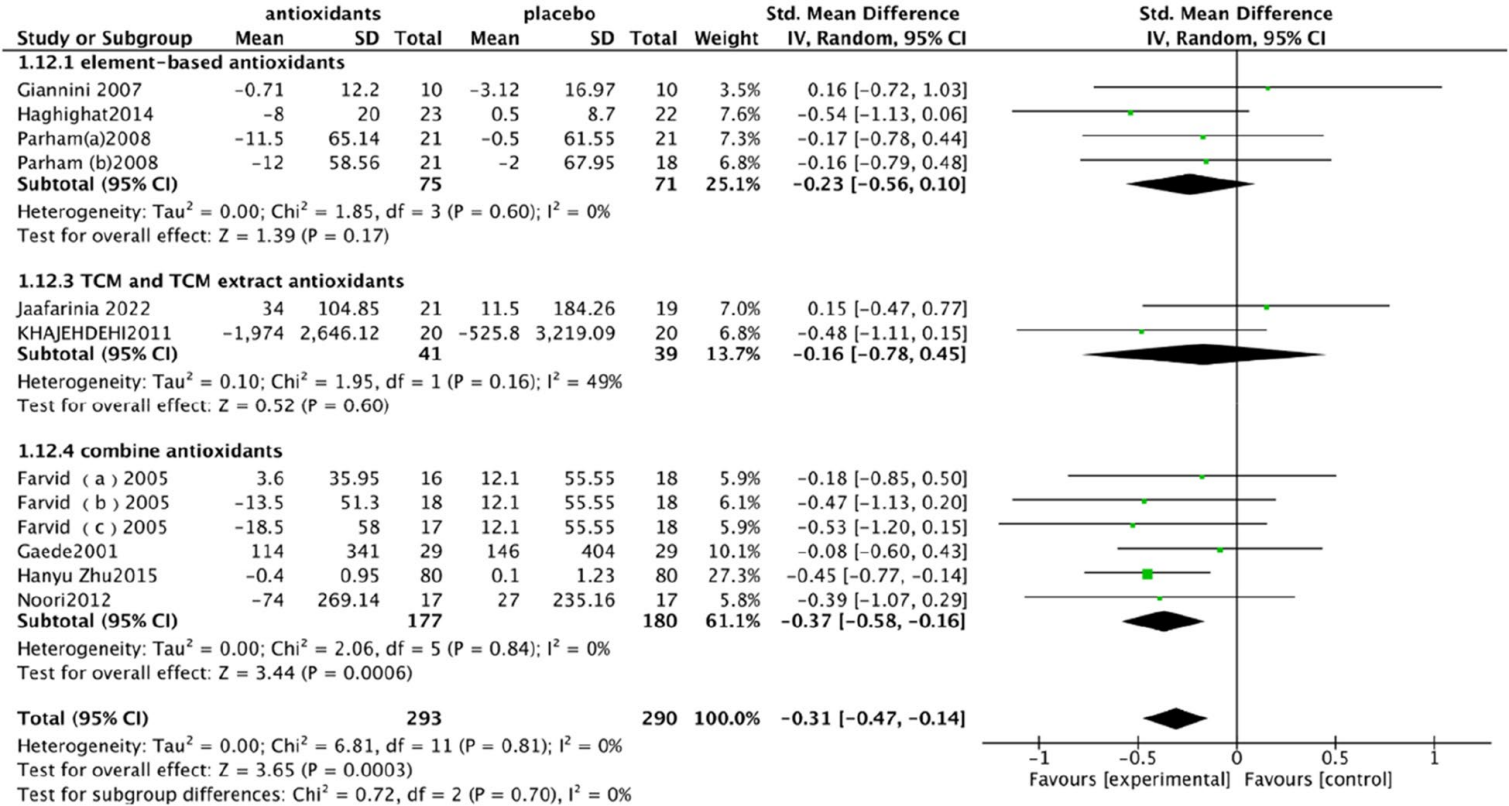
The differences in the effects of trace element-based antioxidants, TCM and TCM extract antioxidants and combine antioxidants

The antioxidants included in this article are different types of antioxidants, including element-based antioxidants, TCM and TCM extract antioxidants and combine antioxidants. In order to analyze the effect of different types of antioxidants on DKD, the main index of antioxidant influence was subgroup analysis, and the results of the element-based antioxidants group showed Chi^2^ = 1.85, df = 3 (P = 0.60), I^2^ = 0%, the test for overall effect: Z: 1.39 (P = 0.17). The results of TCM and TCM extract antioxidants groups showed Chi^2^ = 1.95, df = 1 (P = 0.16), I^2^ = 49%, and the test for overall effect: Z: 0.52 (P = 0.60), and the combined antioxidants group showed Chi^2^ = 2.06, df = 5 (P = 0.84), I^2^ = 0% and the test for overall effect: Z: 3.44 (P = 0.0006) (Fig. 3). According to the results, the effects of different types of antioxidants on UAE were different, showing that the combined application of antioxidants had the best control effect on UAE, while TCM and TCM extracts had heterogeneity. As TCM and TCM extracts were included in fewer studies, more studies are needed to validate. Whether the combination has the best therapeutic effect on DKD also needs to be further verified.

### Secondary outcomes

**Fig. 4:**
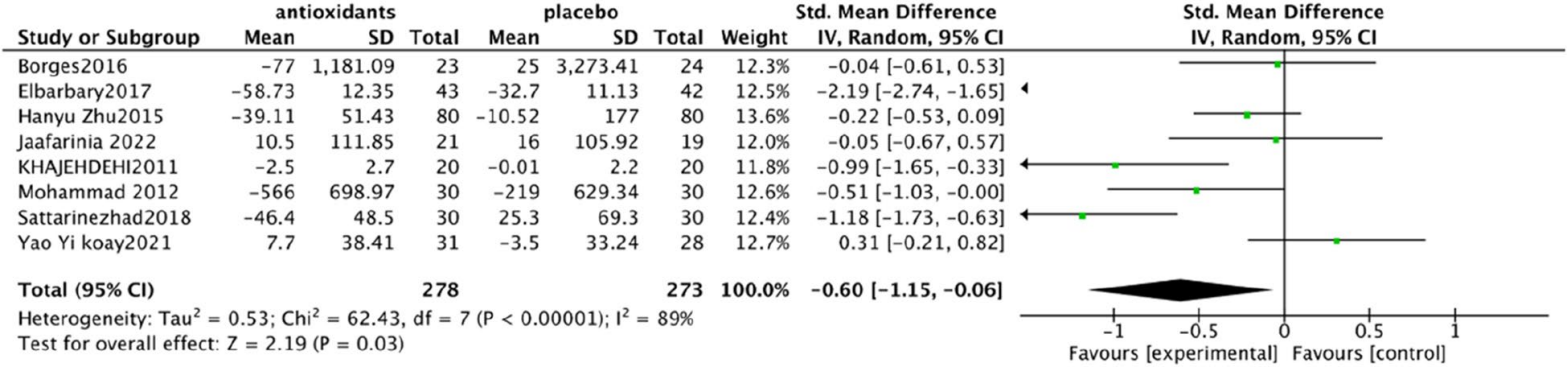
Effect of antioxidants vs. control on urine albumin/creatinine ratio (UACR)

**Fig. 5:**
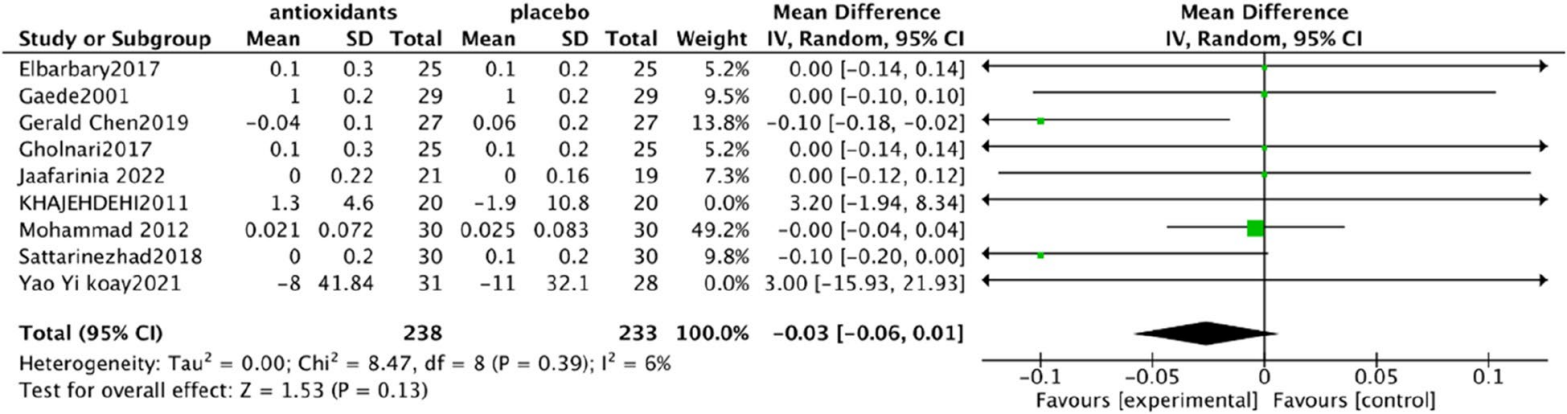
Effect of antioxidants vs. control on serum creatinine (SCr)

**Fig. 6:**
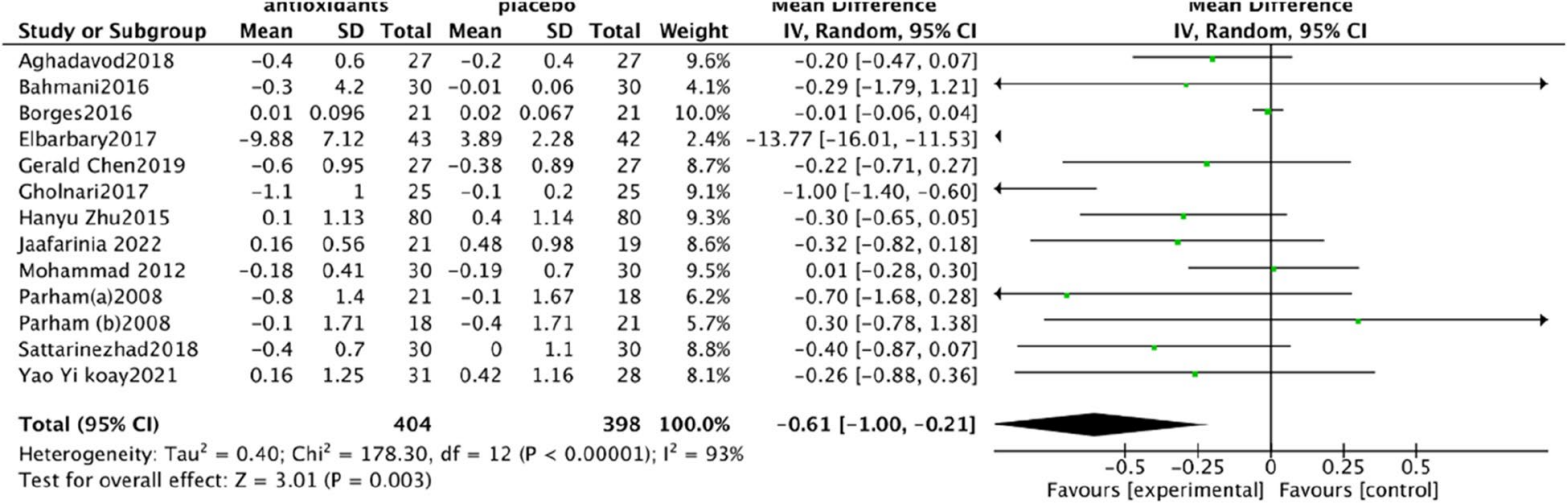
Effect of antioxidants vs. control on glycated hemoglobin glycosylated hemoglobin (hbA1c)

**Fig. 7:**
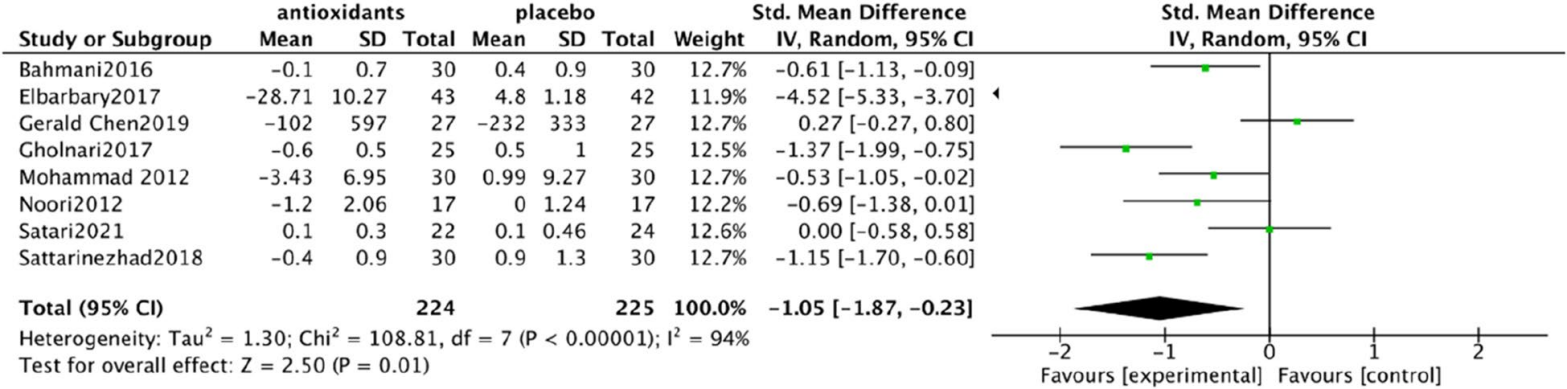
Effect of antioxidants vs. control on Malonaldehyde (MDA)

1. Urine albumin/creatinine ratio (UACR). In a pooled analysis of 8 studies [93, 94, 97, 98, 101, 102, 106, 108], the use of antioxidants was associated with a significant reduction in UACR levels compared with placebo (SMD: − 0.60; 95% CI [− 1.15, − 0.06], Fig. 4). The analysis had high heterogeneity (Chi^2^ = 62.43, df = 7 (P < 0.00001); I^2^ = 89%). The Test for overall effect: Z: 2.19 (P = 0.03).
2. Serum creatinine (SCr). In a pooled analysis of 9 studies [94, 95, 97, 98, 102, 104–106, 108], antioxidant use did not significantly improve Scr levels compared with placebo (MD: − 0.03; 95% CI [− 0.06, 0.01], Fig. 5). The heterogeneity of this analysis was low (Chi^2^ = 8.47, df = 8 (P = 0.39); I^2^ = 6%). Test for overall effect: Z:1.53 (P = 0.13).
3. Glycated hemoglobin glycosylated hemoglobin(hbA1c). In a pooled analysis of 12 studies [92–94, 98, 100–106, 108], the use of antioxidants was associated with a significant reduction in hbA1c levels compared with placebo (MD: − 0.61; 95% CI [− 1.00, − 0.21], Fig. 6). The analysis had high heterogeneity (Chi^2^ = 178.30, df = 12 (P < 0.00001); I^2^ = 93%). The Test for overall effect: Z: 3.01 (P = 0.003).
4. Malonaldehyde(MDA). In a pooled analysis of 8 studies [98–100, 102, 104–107], antioxidants use was associated with a significant reduction in MDA levels compared with placebo (SMD: − 1.05; 95% CI [− 1.87, − 0.23], Fig. 7). The analysis had high heterogeneity (Chi^2^ = 108.81 df = 7(P < 0.00001); I^2^ = 94%). The Test for overall effect: Z: 2.50 (P = 0.01).

### Subgroup analysis

**Fig. 8:**
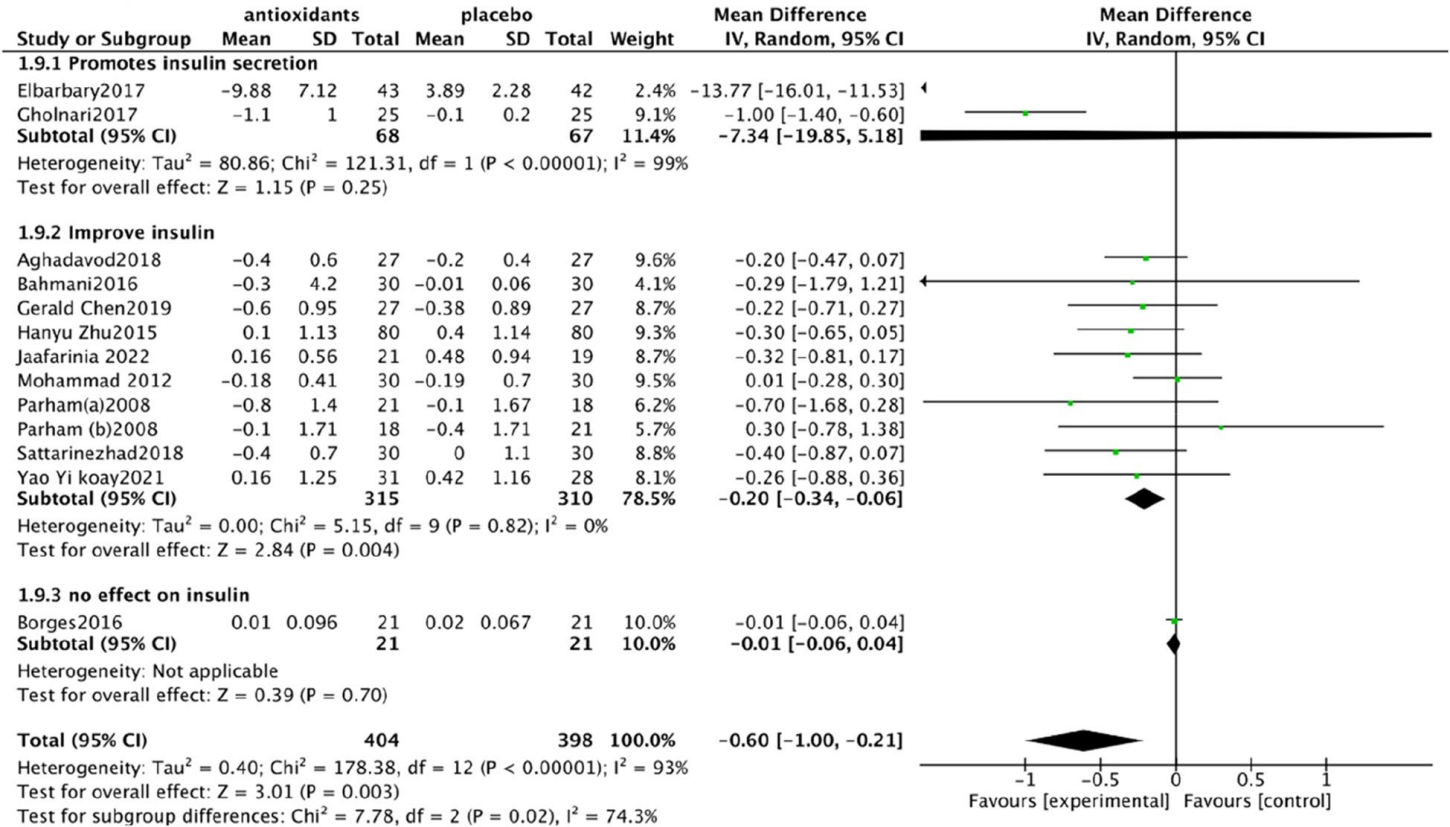
HbA1c subgroup analysis

**Fig. 9:**
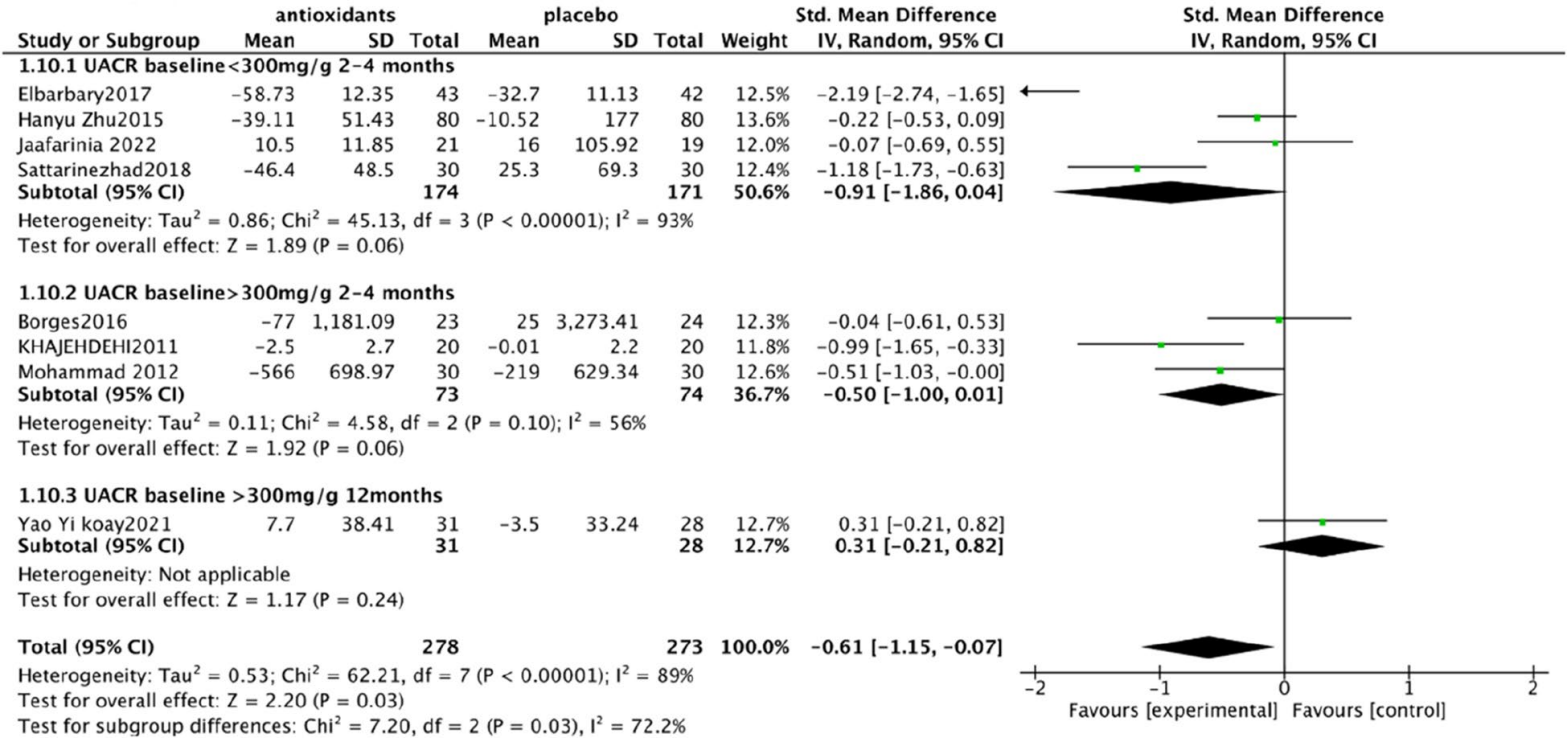
UACR subgroup analysis

1. HbA1c subgroup analysis. Due to the high heterogeneity of hbA1c, I^2^ = 93%, subgroup analysis was performed. Studies have shown that coenzyme Q10 and carnosine can increase the level of insulin, carnosine can promote the secretion of insulin [97], and coenzyme Q10 can promote the synthesis and secretion of insulin [109]. Vitamin E, selenium, zinc, resveratrol, silymarin, probucol, and crocin can help improve insulin, vitamin E can improve insulin resistance and insulin sensitivity [110], selenium can improve insulin sensitivity [111], zinc can regulate insulin receptor, which is an insulin-like substance [112, 113], silymarin can improve insulin resistance [114], and resveratrol can increase insulin sensitivity [115] and improve insulin resistance. Probucol can improve blood glucose levels in insulin-resistant mouse models [116]. Crocin can improve insulin resistance and increase insulin sensitivity [90]. However, there is no clear evidence that green tea polyphenols affect insulin levels [117]. These results indicate that different types of antioxidants have different effects on insulin and hbA1c, suggesting that we should consider the selection of antioxidants in the process of use. Therefore, antioxidants were divided into three groups for subgroup analysis. After analysis, heterogeneity changed in the three groups. In the coenzyme Q10 and carnosine groups, heterogeneity was higher I^2^ = 99%, which may be significantly related to the reduction of hbA1c in carnosine. Heterogeneity was significantly reduced in the use of vitamin E, selenium, zinc, resveratrol, silymarin, crocin, and probucol group, I^2^ = 0% ( Fig. 8).
2. UACR subgroup analysis. Due to the high heterogeneity of UACR, I^2^ = 89%, subgroup analysis was performed. DKD staging was proposed according to KDIGO guidelines and the expert consensus of the Chinese Society of Endocrinology [118], and GA staging was adopted. A represents proteinuria level, divided into A1-3 (A1, UACR < 30 mg/g; A2, UACR 30-300 mg/g; A3, UACR > 300 mg/g). The included studies were grouped into phase A2 studies using carnosine, resveratrol, crocin, and probucol. The phase A3 studies used green tea polyphenols, turmeric, silymarin, and vitamin E. In A3, green tea polyphenols, turmeric, and silymarin delivery time in 2–4 months, however, vitamin E dosing time lasted 12 months, in the use of vitamin E [106] in the research of subgroup analysis showed that before for eight months and can obviously improve the serum creatinine, urine protein. Therefore, patients belonging to stage A3 will continue to be grouped, according to the intervention of green tea polyphenols, turmeric, and silymarin for 2–4 months and vitamin E for 12 months. The heterogeneity was reduced by subgroup analysis of three groups. In the carnosine, resveratrol, crocin, and probucol groups, heterogeneous glue was high, I^2^ = 93%, which may be significantly associated with carnosine lowering UACR. Heterogeneity was reduced in the green tea, turmeric, and silymarin groups, with I^2^ = 56%, possibly associated with different baseline levels of UACR. This subgroup suggests that the clinical use of antioxidants should be based on the stage of DKD, which is closely related to efficacy and prognosis (Fig. 9).
3. MDA. According to the results, the heterogeneity of MDA decreased by antioxidants was relatively high, which may be related to the different antioxidants capacity and the different baseline levels of ACR (urinary albumin to creatinine ratio) in the population, thus leading to the individual differences in MDA.

### Publication bias

**Fig. 10:**
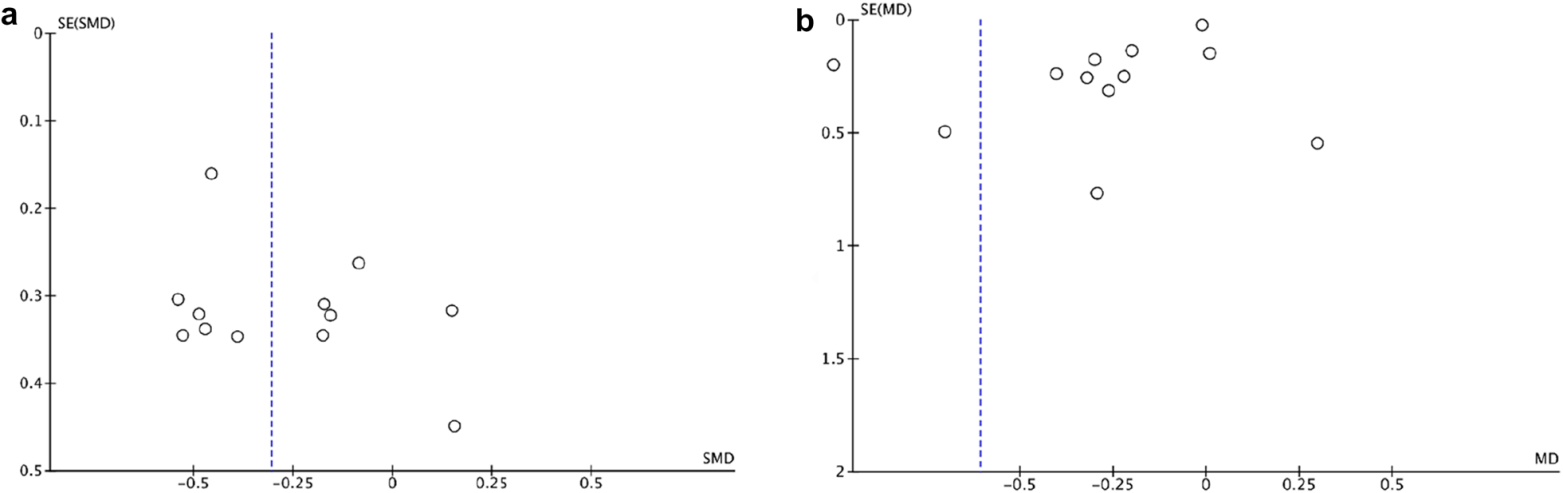
Publication bias. Publication bias was assessed for UAE (**a**) and hbA1c (**b**)

Publication bias is represented by the funnel plot. Publication bias was assessed for UAE and hbA1c because 10 or more trials were included for publication bias. There is no obvious asymmetry in the UAE funnel plot. There was some asymmetry in the funnel plot of hbA1c, indicating a potential publication bias. Unpublished studies may be considered a factor in publication bias (Fig. 10).

### Risk of bias

The risk of RCT bias is summarized in the table (Table 3). Fourteen studies provided information on generating randomized sequences [90, 92–94, 96, 98, 100–106, 108]. Fourteen studies improved assignment hiding methods [89, 92, 94, 96–98, 100–103, 105–108], and eighteen randomized controlled trials were double-blind [89, 90, 92–107] and one trial was triple-blind [108]. All studies had low attrition bias [89, 90, 92–108]. The reporting bias was low in one study and unclear in the remaining subjects [90]. In one study [105], the measurement results were not accurate enough to be considered high-risk and no other source of bias was identified.

**Table 3:** Risk bias included studies

Study, year	Random sequence generation	Allocation concealment	Blinding of participants and personnel	Blinding of outcome assessors	Incomplete outcome data	Selective reporting	Other sources of bias
Gaede et al. (2001) [95]	Unclear (not stated)	Unclear (not stated)	Low risk (double blind)	Unclear (not stated)	Low risk (no drop-out)	Unclear (not stated)	Unclear (not stated)
Farvid et al. (2005) [96]	Low risk (block randomization procedure)	Low risk (the supplement and placebo capsules looked identical)	Low risk (double blind)	Unclear (not stated)	Low risk (two drop-out)	Unclear (not stated)	Unclear (not stated)
Giannini et al. (2007) [89]	Unclear (not stated)	Low risk (Vitamin E and placebo were capsules of the same size, shape, and color)	Low risk (double blind)	Unclear (not stated)	Low risk (no drop-out)	Unclear (not stated)	Unclear (not stated)
Parham et al. (2008) [92]	Low risk (card-shufflfling randomization)	Low risk (the same as the zinc capsules, in size, shape and color.)	Low risk (double blind)	Unclear (not stated)	Low risk (eight drop-out: four in the control group; four in the intervention group)	Unclear (not stated)	Unclear (not stated)
Khajehdehi et al. (2011) [97]	Unclear (not stated)	Low Risk (three capsules identical in colour and size, containing starch)	Low risk (double blind)	Unclear (not stated)	Low risk (no drop-out)	Unclear (not stated)	Unclear (not stated)
Fallahzadeh et al. (2012) [98]	Low risk (sequence generated by Random Allocation Software)	Low Risk (similar in size, shape, weight, color, and taste.)	Low risk (double blind)	Unclear (not stated)	Low risk (four drop-out: two in the control group; two in the intervention group)	Unclear (not stated)	Unclear (not stated)
Noori et al. (2013) [99]	Unclear (not stated)	Unclear (not stated)	Low risk (double blind)	Unclear (not stated)	Low risk (no drop-out)	Unclear (not stated)	Unclear (not stated)
Haghighat et al. (2014) [90]	Low risk (participants were assigned into two groups randomly by using a random number table)	Unclear (not stated)	Low risk (double blind)	Unclear (not stated)	Low risk (five drop-out: three in the control group; two in the intervention group)	Low risk (all the specifified outcomes have been reported)	Unclear (not stated)
Zhu et al. (2016) [93]	Low risk (randomization sequence was created using SAS version 9.2	Unclear (not stated)	Low risk (double blind)	Unclear (not stated)	Low risk (20 drop-out: 13 in the control group; 7 in the intervention group)	Unclear (not stated)	Unclear (not stated)
Bahmani et al. (2016) [100]	Low risk (computer- generated random Numbers)	Low Risk (placebo capsules (starch), including colour, shape, size and packaging, was identical to Se capsules)	Low risk (double blind)	Low risk (concealed from the researchers and participants until the fifinal analyses were completed.)	Low risk (eight drop-out: four in the control group;four in the intervention group)	Unclear (not stated)	Unclear (not stated)
Borges et al. (2016) [101]	Low risk (website Randomization.com was used to Generate)	Low Risk (All drugs and placebo tablets were similar in size, shape, weight, and color.)	Low risk (double blind)	Unclear (not stated)	Low risk (five drop-out: three in the control group; two in the intervention group)	Unclear (not stated)	Unclear (not stated)
Elbarbary et al. (2018) [102]	Low risk (computer-generated randomization sequence)	Low risk (drug pharmacy with allocation concealment by opaque sequentially numbered sealed envelope)	Low risk (double blind)	Unclear (not stated)	Low risk (five drop-out: three in the control group;two in the intervention group)	Unclear (not stated)	Unclear (not stated)
Aghadavod et al. (2018) [103]	Low risk ( using a random number table)	Low Risk (similar in shape and size to vitamin E capsule)	Low risk (double blind)	Unclear (not stated)	Low risk (six drop-out: three in the control group; three in the intervention group)	Unclear (not stated)	Unclear (not stated)
Gholnari et al. (2018) [104]	Low risk (computer-generated random numbers)	Unclear (placebo (cellulose))	Low risk (double blind)	Unclear (not stated)	Low risk (three drop-out in intervention group)	Unclear (not stated)	Unclear (not stated)
Tan et al. (2019) [105]	Low risk (computer-generated random sequence)	Low Risk (investigational products was kept Confidential)	Low risk (double blind)	Unclear (not stated)	Low risk (no drop-out)	Unclear (not stated)	High Risk (tocotrienol measurements were not accurate.)
Sattarinezhad et al. (2019) [106]	Low risk (randomized list generated by Microsoft Excel software)	Low Risk (identical shapes, sizes and colours of the resveratrol and placebo containers)	Low risk (double blind)	Unclear (not stated)	Low risk (four drop-out: two in the control group; two in the intervention group)	Unclear (not stated)	Unclear (not stated)
Satari et al. (2021) [107]	Unclear (not stated)	Low Risk (Melatonin and placebo capsules were produced in the same shape and Package)	Low risk (double blind)	Unclear (not stated)	Low risk (eight drop-out: four in the control group; four in the intervention group)	Unclear (not stated)	Unclear (not stated)
Koay et al. (2021) [94]	Low risk (computer-generated random sequence)	Low risk (identical looking capsules (tocotrienol-free palm oil capsules)	Low risk (double blind)	Unclear (not stated)	Low risk (two drop-out)	Unclear (not stated)	Unclear (not stated)
Jaafarinia et al. (2022) [108]	Low risk (Microsoft Excel software with a block randomization method)	Low risk (Both intevention and placebo tablets were similar in size, shape, weight, and color)	Low risk (triple-blind)	Low risk (triple-blind)	Low risk (four drop-out: three in the control group; one in the intervention group)	Unclear (not stated)	Unclear (not stated)

The quality of each article was independently assessed using the Cochrane bias risk assessment tool

### Adverse events

Thirteen studies reported no significant adverse events [89, 90, 92, 95–97, 99, 100, 102–104, 106, 107]. In the study of Borges et al. one patient had diarrhea after GTP intervention, one patient had dyspepsia, and one patient had dizziness in the placebo group [101]. In the study by Jaafarinia et al. there was one tremor in the saffron intervention group and one dysuria in the placebo control group [108]. In the study of Tan et al. a total of twenty-eight adverse events were reported, including thirteen in the intervention group and fifteen in the control group [105]. In the intervention group, one patient developed septic shock secondary to bronchopneumonia three days after admission, and one patient developed cerebrovascular events (left forebrain infarction). In the control group, one patient developed septic shock secondary to left leg cellulitis. In Fallahzadeh et al. study, a total of nine adverse events were reported [98], including one serious adverse event, one patient died of myocardial infarction in the silymarin intervention group, six patients in the intervention group and two in the placebo group, and three patients experienced nausea and vomiting during silymarin treatment. Headache occurred in two patients, and dyspepsia and abdominal distension occurred in one patient. Two patients in the placebo group reported nausea and vomiting. In the study of Koay et al. three patients had adverse events unrelated to the intervention [94]. In the study of Zhu et al. a total of ten adverse events were reported, and five adverse events were reported in the intervention group and the control group, with a total of four serious adverse events [93]. In the telmisartan placebo group, one patient was reported to have myocardial infarction and one patient was reported to have a stock. One death and one heart failure were reported in the telmisartan + probucol intervention group. In the placebo group, two patients had hyperkalemia and one patient had liver insufficiency. In the intervention group, there were two cases of hyperkalemia and one case of hypertension.

## Discussion

### Summarize evidence and explain outcomes

In recent years, the incidence of DKD has increased significantly with the increase of the incidence of diabetes, and metformin is the first choice and basic drug for T2DKD patients to control blood glucose [118]. ACEI/ARB (angiotensin-converting enzyme inhibitor/angiotensin II receptor antagonist) [118] is the preferred treatment for T2DKD microalbuminuria. ADA and KDOQI guidelines recommend ACEI/ARB as first-line therapy for T2DKD in patients with proteinuria [6, 97]. However, the current study does not suggest that both are effective in controlling blood glucose and improving proteinuria. Clinical trials have shown that antioxidants can both control blood sugar and improve proteinuria as antioxidant use has increased. This study was a meta-analysis and systematic review to determine the efficacy and safety of antioxidant therapy for DKD. Of the 554 RCTs for possible inclusion, 19 studies met the inclusion criteria. Results showed a positive effect of antioxidants in the treatment of DKD patients, with outcomes including improvement of UAE, UCAR, and reduction of hbA1c and MDA compared to the control group. Pathological UAE is one of the strongest and earliest signs of kidney damage caused by diabetes. The abnormality was initially caused by impaired glomerular filtration barrier, which increased plasma protein permeability [112]. HbA1c is considered a gold indicator of blood glucose control and is closely related to DKD [119]. However, the results of this study did not show a significant improvement effect on SCr, which may be related to the short intervention time and small sample size. In this meta-analysis, hbA1c, UCAR, and MDA showed high heterogeneity. After subgroup analysis, the heterogeneity was significantly reduced, suggesting that the type of antioxidants and the patient's baseline level may influence the treatment effect, and may also be related to the short intervention time and small sample size. Of the 19 studies, 13 reported no adverse events and the remaining six reported adverse events, no adverse events were reported due to antioxidants use compared to the control group, and there were no significant differences in the incidence of adverse events between the intervention and control groups, suggesting that antioxidants seem safe in the treatment of DKD.

### Limitations

The main limitation is the small sample size. Only one study had 160 participants, and the others had smaller samples. Most trials lasted 12 weeks, and improvements in kidney function may not be better assessed without longer intervention and observation. The existing studies and sample sizes are slightly inadequate. Due to certain deficiencies in the duration, staging and efficacy evaluation of clinical trials, the conclusions reached are relatively weak. At the same time, many antioxidants are being tested in clinical trials for the first time, and there is no additional evidence to support their efficacy. In addition, some of the statistics included in the included studies were biased and could lead to imprecision in the analyses.

### Impact of the study

Restoration of the balance between oxidative stress and antioxidant defense may be a potential drug target for the prevention and treatment of DKD [61]. Diabetes-induced ROS drives the thickening of the glomerular basement membrane, mesangial dilation, accumulation of extracellular matrix, glomerular sclerosis, and abnormal renal hemodynamics [112]. The use of antioxidants has brought positive significance to DKD patients. It can be seen from the results that antioxidants can reduce blood glucose and improve proteinuria, which provides a good direction and idea for the clinical treatment of DKD and lays a good foundation for further clinical trials and more basic studies.

### Summary

This article reviews the relationship between oxidative stress and antioxidants and DKD, demonstrating the mechanism by which oxidative stress is involved in DKD, and then our systematic review and meta-analysis results indicate that antioxidants appear to have therapeutic benefits in patients with DKD, especially in improving proteinuria and reducing hbA1c. However, the number of existing studies is insufficient, including sample size and drug replication studies, and larger randomized controlled trials are needed. The results of this study provide a good direction for the clinical treatment of patients with DKD.

## Supplementary Information

**Additional file 1.** Search strategy of Pubmed.

## Data Availability

Not applicable.

## Abbreviations

DKD: Diabetic kidney disease
UAE: Albumin excretion rate
UACR: Urine albumin/creatinine ratio
hbA1c%: Glycosylated hemoglobin
MDA: Malonaldehyde
CKD: Chronic kidney disease
ESRD: End-stage renal disease
GFR: Glomerular filtration rate
NOX1: NADPH oxidase 1
IL-6: Interleukin 6
MCP-1: Monocyte chemoattractant protein-1
TGF-β: Transforming growth factor-β
NADPH: Nicotinamide adenine dinucleotide phosphate
VEGF: Vascular endothelial growth factor
GBM: Glomerular basement membrane
NF-κB: Nuclear factor κB
TNF-α: Tumor necrosis factor-alpha
CAMs: Cell adhesion molecules
MAPK: Mitogen-activated protein kinase
AP-1: Transcription factor activator protein 1
JNK: C-Jun N-terminal kinase
OS: Overall survival
NO: Nitric oxide
AGEs: Advanced glycation end products
PKC: Protein kinase C
ECM: Extracellular matrix
CTGF: Connective tissue growth factor
TGF-β1: Transforming growth factor-β1
Nrf2: Nuclear factor (erythroid-derived 2)-like 2
HO-1: Hemoglobin oxygenase-1 (hypocretin-1)
NQO1: Quinone oxidoreductase (NAD(P)H Quinone Dehydrogenase 1)
CAT: Catalase
SOD: Superoxide dismutase
GPx: Glutathione peroxidase
NR1: Notoginsenoside R1
HFD: High-fat diet
STZ: Streptozotocin
HSYA: Hydroxyl safflower yellow A
GSH-Px: Glutathione
RAGE: Receptor for advanced glycosylation end products
NFE2L2: Nuclear factor erythroid 2-related factor 2
HMOX1: Heme oxygenase-1
SLC30A7: The 7th member of SLC30 family
SGLT2: Sodium/glucose co-transporter type 2
Sp1: Specificity protein 1
TLR4: Toll-like receptor 4
PGC-1α: Proliferator-activated receptor γ coactivator-1α
GCN5L1: General control of amino acid synthesis 5-like 1
MnSOD: Manganese superoxide dismutase
LGR6: Leucine-rich repeat domain-containing G protein-couple receptor 6
cAMP: Cyclic adenosine 3’,5’ -monophosphate
SOD2: Mn-SOD
CuNPs: Hydrogen sulfide
GST: Glutathione S transferase
RCTs: Randomized controlled trials
SCr: Serum creatinine
MD: Mean difference
SMD: Standard mean difference
ACEI: Angiotensin-converting enzyme inhibitor
ARB: Angiotensin II receptor antagonist
ROS: Reactive oxygen species
