# Supplementary material for: Effects of antioxidants on diabetic kidney diseases: mechanistic interpretations and clinical assessment
Source: Chin Med. 2023 Jan 9;18:3. doi: 10.1186/s13020-022-00700-w (PMC9827645; doi:10.1186/s13020-022-00700-w)
Supplement: Supplementary file 1 — Additional file 1. Search strategy of Pubmed. [file 13020_2022_700_MOESM1_ESM.docx]

File 1, Search strategy of Pubmed

2022-2011

1. Antioxidants[Title/Abstract]

2. Anti-Oxidants[Title/Abstract]

3. Anti Oxidants[Title/Abstract]

4. Antioxidant[Title/Abstract]

5. Anti-Oxidant[Title/Abstract]

6. Anti Oxidant[Title/Abstract]

7. Antioxidant Activity[Title/Abstract]

8. Activity, Antioxidant[Title/Abstract]

9. Antioxidant Effect[Title/Abstract]

10. Anti-Oxidant Effect[Title/Abstract]

11. Anti Oxidant Effect[Title/Abstract]

12. Anti-Oxidant Effects[Title/Abstract]

13. Anti Oxidant Effects[Title/Abstract]

14. Antioxidant Effects[Title/Abstract]

15. 1 or 2 or 3 or 4 or 5 or 6 or 7 or 8 or 9 or 10 or 11 or 12 or 13 or 14

16. Diabetic Nephropathy[Title/Abstract]

17. Diabetic Kidney Disease[Title/Abstract]

18. Diabetic Kidney Diseases[Title/Abstract]

19. Kidney Disease, Diabetic[Title/Abstract]

20. Kidney Diseases, Diabetic[Title/Abstract]

21. Diabetic Glomerulosclerosis[Title/Abstract]

22. Glomerulosclerosis, Diabetic[Title/Abstract]

23. Intracapillary Glomerulosclerosis[Title/Abstract]

24. Nodular Glomerulosclerosis[Title/Abstract]

25. Glomerulosclerosis, Nodular[Title/Abstract]

26. Kimmelstiel-Wilson Syndrome[Title/Abstract]

27. Kimmelstiel Wilson Syndrome[Title/Abstract]

28. Syndrome, Kimmelstiel-Wilson[Title/Abstract]

29. Kimmelstiel-Wilson Disease[Title/Abstract]

30. Kimmelstiel Wilson Disease[Title/Abstract]

31. Nephropathies, Diabetic[Title/Abstract]

32. Nephropathy, Diabetic[Title/Abstract]

33. 16 or 17 or 18 or 19 or 20 or 21 or 22 or 23 or 24 or 25 or 26 or 27 or 28 or 29 or 30 or 31 or 32

33. radomized controlled trial [Publication Type]

34. controlled clinical trial [Publication Type]

35. radomized [Tittle/Abstract]

36. controlled [Tittle/Abstract]]

37. trial [Tittle]

38. radom [Tittle/Abstract]

39. placebo [Tittle/Abstract]

40. groups [Tittle/Abstract]

41. 33 or 34 or 35 or 36 or 36 or 37 or 38 or 39 or 40

42. 15 and 33 and 41
